# A novel mitochondrial-related lncRNA signature mediated prediction of overall survival, immune landscape, and the chemotherapeutic outcomes for bladder cancer patients

**DOI:** 10.1007/s12672-024-01108-8

**Published:** 2024-06-21

**Authors:** Hao Xiong, Cheng Lin, Xiang Huang, Hao Wang

**Affiliations:** 1 Department of Urology, Nanping First Hospital Affiliated to Fujian Medical University, Nanping, 353000 China; 2 Department of Urology, The First Affiliated Hospital, Hengyang Medical School, University of South China, Hengyang, 421001 China

**Keywords:** Bladder cancer, Mitochondrial dysfunction, Prognostic risk model, Immune landscape, Chemotherapeutic

## Abstract

**Objective:**

To develop a prognostic risk model for Bladder Cancer (BLCA) based on mitochondrial-related long non-coding RNAs (lncRNAs).

**Methods:**

Transcriptome and clinical data of BLCA patients were retrieved from the TCGA database. Mitochondrial-related lncRNAs with independent prognostic significance were screened to develop a prognostic risk model. Patients were categorized into high- and low-risk groups using the model. Various methods including Kaplan–Meier (KM) analysis, ROC curve analysis, Gene Set Enrichment Analysis (GSEA), immune analysis, and chemotherapy drug analysis were used to verify and evaluate the model.

**Results:**

A mitochondrial-associated lncRNA prognostic risk model with independent prognostic significance was developed. High-risk group (HRG) patients exhibited significantly shorter survival periods compared to low-risk group (LRG) patients (P < 0.01). The risk score from the model was an independent predictor of BLCA prognosis, correlating with tumor grade, pathological stage, and lymph node metastasis (P < 0.05). The HRG showed significant positive correlations with high expressions of immune checkpoints (CTLA4, LAG3, PD-1, TIGIT, PD-L1, PD-L2, and TIM-3) and lower IC50 for chemotherapy drugs (cisplatin, docetaxel, paclitaxel, methotrexate, and vinblastine) (P < 0.001).

**Conclusions:**

The mitochondrial-related lncRNA-based prognostic risk model effectively predicts BLCA prognosis and can guide individualized treatment for BLCA patients.

## Introduction

The International Agency for Research on Cancer's GLOBOCAN 2021 report provided estimates for cancer morbidity and mortality rates, revealing that bladder cancer (BLCA) is a prevalent malignant tumor affecting the urinary system, accounting for approximately 7.0% of all new cancer cases. Furthermore, it accounts for about 4.0% of cancer-related deaths, and its morbidity and fatality rates are increasing every year. Thus, BLCA poses a serious threat to human health [1]. Currently, BLCA diagnosis lacks significantly reliable disease prognostic markers and effective molecular therapeutic targets. The primary BLCA treatment is surgery; however, the BLCA recurrence rate is very high. About 50% of the patients relapse and develop metastasis post-radical surgery. In addition, the prognosis is poor. Although the approval of drugs like cisplatin, methotrexate, vinblastine, gemcitabine, and other chemotherapy regimens for patients with advanced BLCA has improved the survival rate of patients with metastatic or unresectable BLCA, the high heterogeneity of BLCA makes the treatment outcomes unsatisfactory [2]. Therefore, it is imperative to develop new prognostic models and molecular markers that can provide more accurate predictions of clinical outcomes among BLCA patients, thereby helping improve patient management and treatment outcomes.

Long non-coding RNAs (lncRNAs) have attracted increasing attention as potential tumor markers for early detection, diagnosis, prognosis, and predicting drug treatment outcomes [2]. LncRNAs have been reported to influence the infiltration of cells, alter the immune microenvironment, and regulate cancer progression. Recently, the involvement of some apoptosis and immune response-regulating lncRNAs in the pathophysiological process of BLCA has been demonstrated [3]. However, it is still unclear whether these involvements originate from disorders and dysregulation of other lncRNAs or from specific regulatory mechanisms. Mitochondria are complex organelles that play crucial roles in bioenergetics and biosynthetic signal transduction. They supply ATP for various cellular processes by oxidative phosphorylation. ATP serves as the energy currency of metabolic pathways, the main source of reactive oxygen species (ROS), and the buffer of intracellular calcium, apoptosis, and signal transduction regulators. Recently, mitochondrial genome instability and mitochondrial dysfunction have been reported as new markers of cancer [4]. Hence, some specific nuclear mitochondrial genes (NMGs) are considered potential targets for the development of next-generation cancer therapies [5]. It is noteworthy that lncRNAs can regulate mitochondrial function, thus affecting cell survival. Previous studies have shown that mitochondria-related lncRNA RMRP is highly expressed in bladder cancer tissues. Additionally, the expression of RMRP is closely related to the patient's tumor size, lymph node metastasis, and survival time. More importantly, RMRP can promote the proliferation, migration, and invasion of BC cell lines by regulating miR-206 like a sponge [6]. Recent studies have demonstrated that aerobic glycolysis is the primary energy source that sustains uncontrolled cell growth and proliferation in BLCA. Therefore, BLCA cells exhibit the overexpression of genes encoding glycolysis, the pentose phosphate pathway, and fatty acid synthesis, suggesting that mitochondrial activity is suppressed in this cancer [7].

We hypothesize that specific mitochondrial-related lncRNAs can serve as reliable prognostic markers for BLCA. By systematically analyzing their expression and clinicopathological features, we aim to develop a prognostic risk model that not only predicts patient outcomes but also informs treatment decisions, including chemotherapy and immunotherapy responses. In this study, we systematically analyzed the relationship between mitochondrial-associated lncRNA expression and clinicopathological features in 409 BLCA patients from the TCGA database. We identified 6 mitochondrial-associated lncRNAs to establish a BLCA prognosis risk model. Moreover, the independent potential of these six selected lncRNAs in accurately predicting BLCA patients’ prognoses, along with their sensitivity towards chemotherapeutic drugs and immunotherapy, was evaluated.

## Materials and methods

### Clinical data

The RNA transcriptome as well as corresponding clinical data of BLCA patients have been retrieved utilizing the TCGA database (https://portal.gdc.cancer.gov/) [8]. The clinical data included survival time, survival status, age, gender, tumor grade, pathological stage, and TNM stages. Thereafter, the FPKM values have been converted to TPM values for the synthetic matrix by applying the following packages (data.table, tibble, dplyr, and tidyr R) The patients who had insufficient clinical data were eliminated. Herein, 401 patients have been classified into the entire set, training set, and testing set.

### Extraction of BLCA mitochondrial-related lncRNA

In total, 147 mitochondrial gene sequences were downloaded from the mitochondrial gene bank. The selection of these genes was based on their documented involvement in mitochondrial function and cancer-related pathways, as indicated by previous studies [9, 10]. A synthetic data matrix was screened using Strawberry Perl and the limma R packages, resulting in the identification of 16876 lncRNAs. Pearson correlation analysis (PCA) was carried out to determine the relationship between lncRNA expression and corresponding mitochondrial-related genes. BLCA mitochondrial-related lncRNAs were extracted based on a correlation coefficient |r|> 0.6 and P < 0.001.

### Construction of the BLCA mitochondrial-related lncRNA prognostic risk model

Among BLCA mitochondrial-associated lncRNAs, prognosis-associated lncRNAs were selected using univariate Cox (uni-Cox) hazard regression analysis. LASSO regression analysis with tenfold cross-validation was then performed to filter the mitochondrial-associated lncRNAs. The LASSO method was used to further screen for mitochondrial-associated lncRNAs with independent prognostic significance through multivariate Cox (multi-Cox) regression analysis and Kaplan–Meier (KM) method. The optimal lncRNA set for the BLCA prognosis risk model was determined using the Akaike Information Criterion (AIC). The Risk Score (RS) was calculated using the following formula: *Risk Score (RS)* = *∑*^*n*^_*k*_ = *1 expression (lncRNA k)* × *coefficient (lncRNA k).*Using the median RS, BLCA patients were segregated into low- and high-risk groups.

### Prognostic risk model analysis

The applicability of the prognostic risk model in clinical settings was evaluated using the Wilcoxon signed-rank test to analyze the clinicopathological characteristics, including survival status, age, sex, tumor grade, clinical stage, and TNM stages among both risk groups. A box plot was used to present the analysis outcomes. The prognostic risk model's ROC curve was constructed to evaluate its specificity and sensitivity, using the area under the curve (AUC). Additionally, univariate and multivariate Cox regression analyses were conducted on the RS and clinicopathological features to identify independent predictors of BLCA prognosis, displayed using forest plot results. To address the risk of model overfitting, bootstrapping methods were employed. Bootstrapping involved repeatedly sampling from the dataset with replacement and assessing the model’s performance on these samples. This method allowed us to estimate the stability and reliability of the prognostic model.

### Gene set enrichment analysis (GSEA)

GSEA software (https://www.gsea-msigdb.org/gsea/login.jsp) [11] has been applied to the curated gene set (kegg.v7.4.symbols.gmt) for identifying the significant pathways across both risk groups, and the identified pathways were enriched based on the criteria of P < 0.05.

### Tumor-infiltrating immune cell examination

Immune cell factors for both risk groups have been evaluated to investigate the correlation between the prognostic risk model and tumor-infiltrating immune cells. Further, the immune infiltration status of BLCA patients was calculated from TCGA on TIMER2.0 (http://timer.cistrome.org/), including seven currently recognized algorithms, namely TIMER [12], CIBERSORT [13], XCELL [14], QUANTISEQ [15], MCPCOUNTER [16], EPIC [17], and CIBERSORT [18]. In addition, TME scores and immune checkpoints among both risk groups have been compared through the ggpubr package in R. The Immune, stromal as well as estimate scores were also computed to characterize the tumor-infiltrating cells.

### Principle component analysis

Generally, PCA is utilized to reduce effective dimensionality, identify models, and visualize groups of mitochondrial-related lncRNAs for the high-dimensional data of global gene expression profiles, mitochondrial genes, and mitochondrial-related lncRNA risk models. KM survival analysis was conducted to assess the diversity of OS between both groups using the survMiner and survival packages in R.

### Sensitivity analysis of immune checkpoint inhibitors (ICIs) and targeted drugs

The levels of ICIs (CTLA4, LAG3, PDL1, PD1, TIGIT, GAL9, TIM-3, and PD1LG2) expression have been evaluated in each BLCA specimen and also compared between both risk groups. The drug's half-inhibition rate (IC50) was utilized as a drug sensitivity indicator. The potency of targeted drugs frequently prescribed for BLCA, such as cisplatin, docetaxel, paclitaxel, methotrexate, and vinblastine was evaluated by measuring their IC50 values. The Wilcoxon signed-rank test has been carried out for determining the differences in IC50 values between both risk groups, and the pRRhetic and ggplot2 packages in R software are applied for results visualization.

### Statistical analysis

The statistical analyses have been conducted utilizing the R software version 4.1.1. The prognostic significance has been evaluated utilizing univariate and multivariate Cox regression analyses as well as Lasso analysis. KM survival curve studies were utilized to analyze overall survival (OS). The reliability, as well as sensitivity of prognostic characteristics, were evaluated through ROC profile analysis and their corresponding AUC values. RS correlation analysis was conducted utilizing the Spearman correlation test. In addition, p < 0.05 was considered a significant difference between all statistics.

## Results

### Screening of mitochondrial-related lncRNA in BLCA

**Fig. 1:**
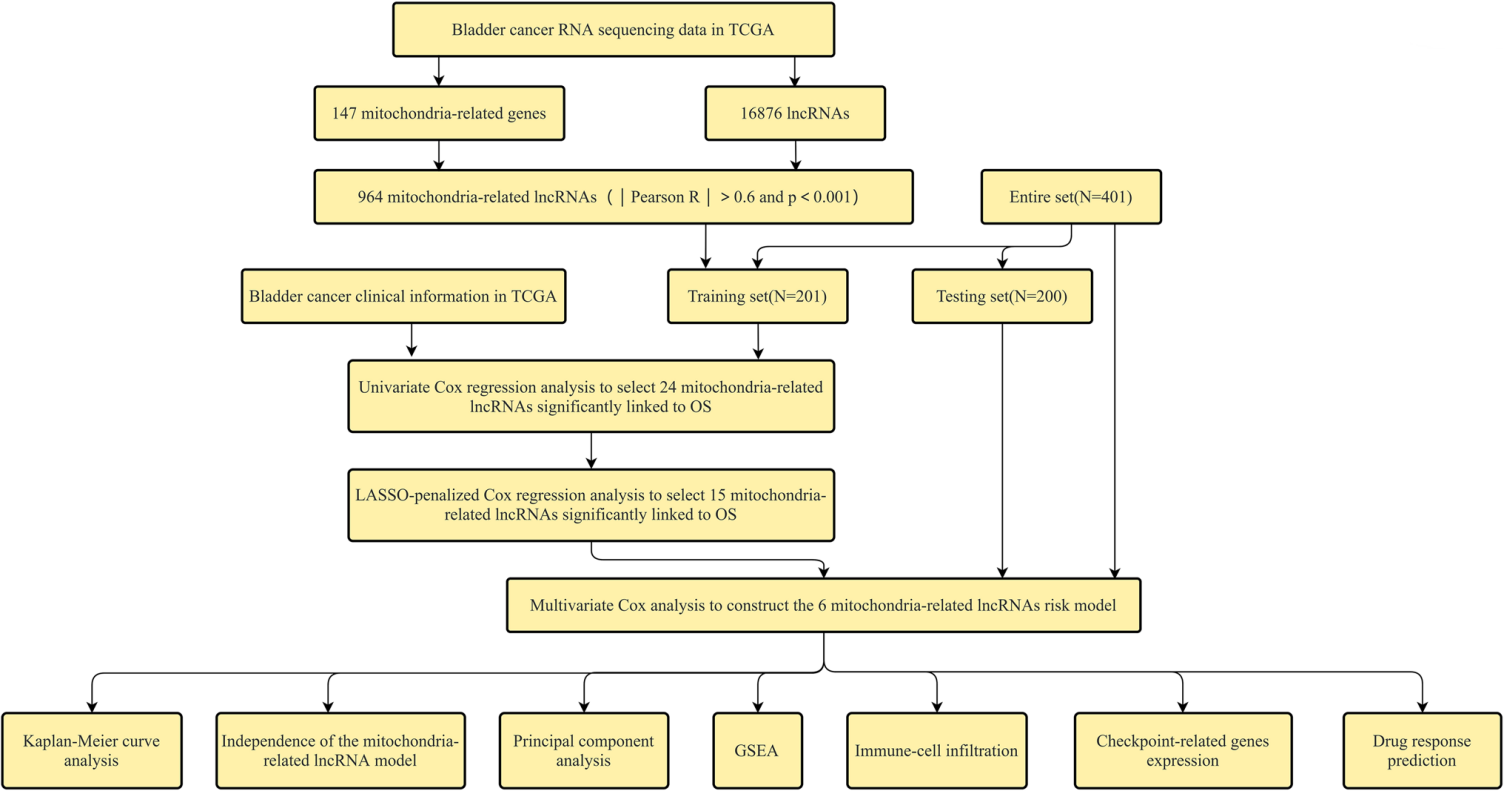
Research flow chart

**Fig. 2:**
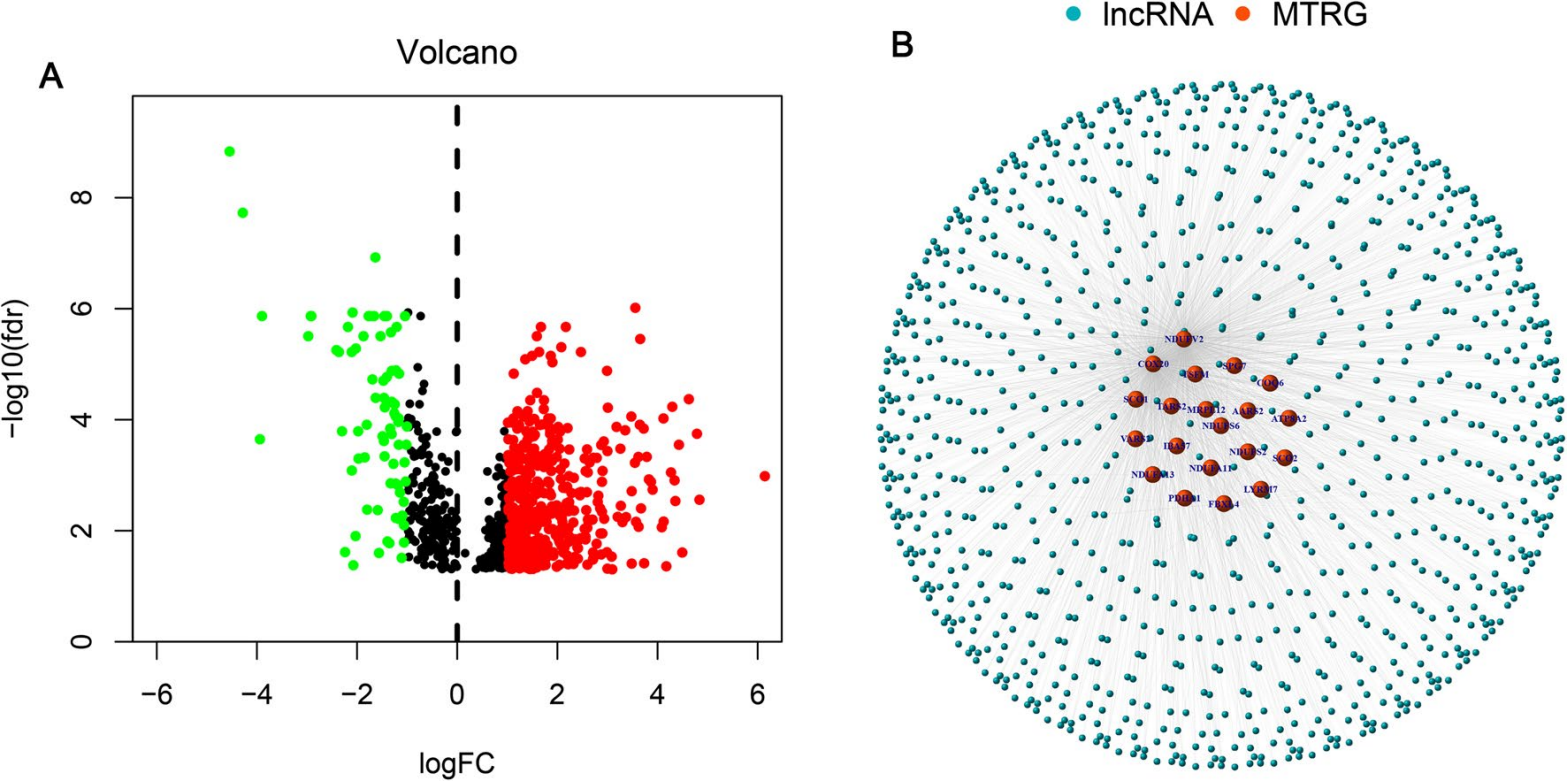
Detection of differentially expressed mitochondrial-related lncRNAs in BLCA patients. **A** Volcano plot of differentially expressed mitochondrial-associated lncRNAs. Green-colored sequences depicting down-regulated lncRNAs, and red-colored for up-regulated lncRNAs. **B** Network constituting mitochondrial genes and lncRNAs

The research scheme is shown in Fig. 1. BLCA raw RNA sequencing data were retrieved from the TCGA database to extract 16,876 lncRNAs, and a co-expression network has been developed with 147 mitochondrial-related coding genes (mRNAs). Thus, a total of 964 mitochondrial-related lncRNAs (correlation coefficient |r|> 0.6 and P < 0.001) was obtained. The limma R package was utilized to perform the differential expression analysis. Consequently, 532 differentially expressed mitochondrial-related lncRNAs (log twofold change (FC) > 1, false discovery rate (FDR) < 0.05) are obtained, and 457 of them underwent up-regulation while 75 underwent down-regulation (Fig. 2A). The network diagram comprising mitochondrial-related genes and lncRNAs is illustrated in Fig. 2B. The identification of differentially expressed mitochondrial-related lncRNAs highlights their potential role in BLCA pathology. Up-regulated lncRNAs may contribute to tumor progression by influencing mitochondrial function, energy metabolism, and reactive oxygen species production, which are crucial for cancer cell survival and proliferation.

### Screening of BLCA prognosis mitochondrial-related lncRNAs and establishment of prognostic risk model

**Fig. 3:**
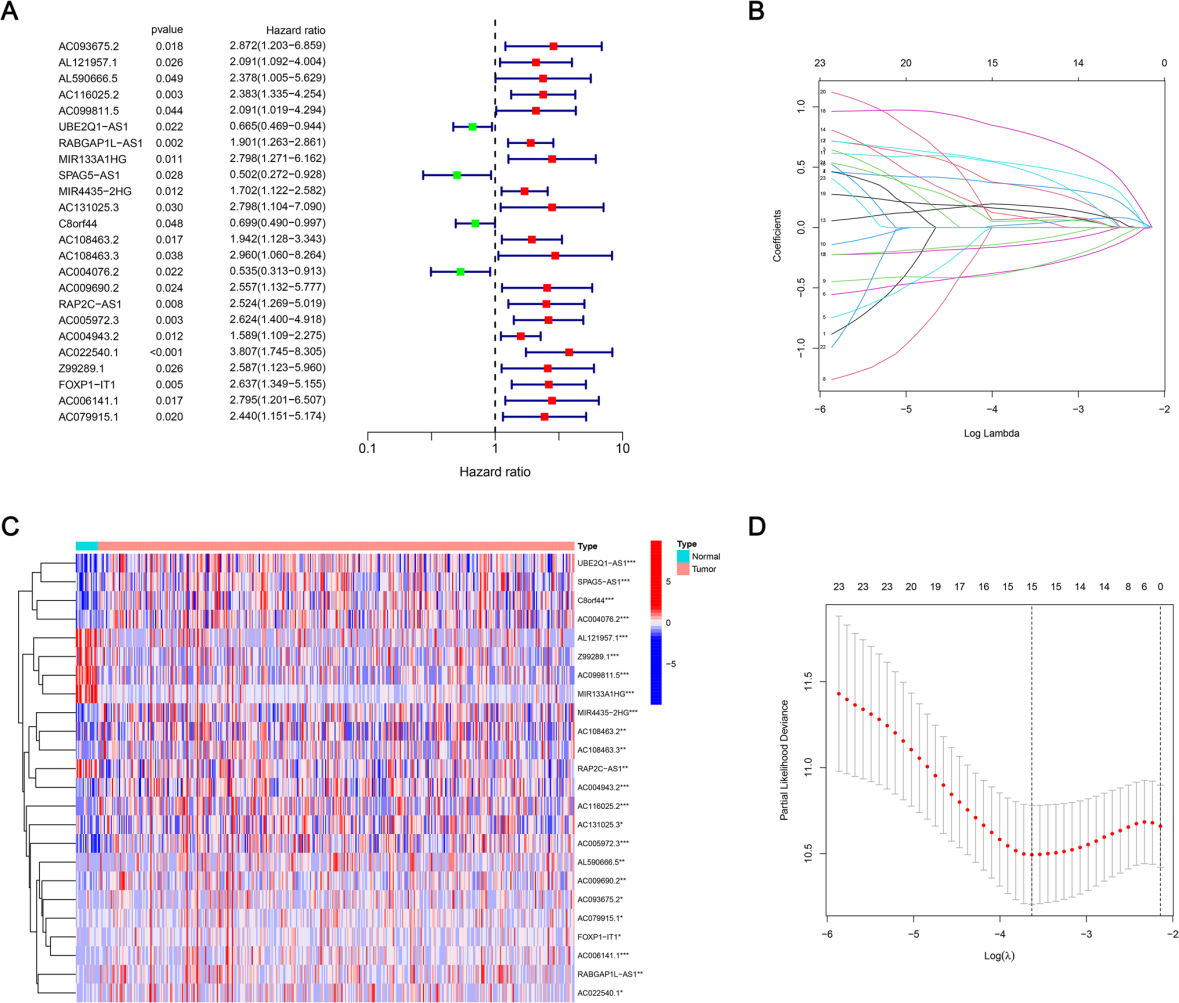
mitochondrial extraction of prognostic features of mitochondrial-associated lncRNAs in BLCA. **A** Mitochondrial-associated prognostic lncRNAs assessed by univariate regression analysis. **B** 24 prognostic mitochondrial-related lncRNAs screened by the LASSO-Cox regression model. **C** Expression profiles of 24 prognostic mitochondrial-related lncRNAs. **D** Distribution of LASSO coefficients of 24 mitochondrial-related lncRNAs

A univariate Cox regression analysis has been executed via R’s “survival” package to calculate the prognostic significance of 532 differentially expressed mitochondrial-related lncRNAs in BLCA (Fig. 3A). 24 lncRNAs showed a considerable association with the BLCA prognosis (P < 0.05) (Fig. 3B). Further, through LASSO analysis, 15 lncRNAs were selected. Multivariate Cox regression analysis and KM method are applied to screen the mitochondrial lncRNAs with independent prognosis, and 6 lncRNAs were determined according to the optimal AIC value to establish a BLCA prognostic risk model (Fig. 3C-D). The selected lncRNAs for the prognostic risk model suggest their independent roles in BLCA progression and patient outcomes. These lncRNAs may regulate key biological processes such as apoptosis, immune response, and mitochondrial function, thereby influencing overall survival.

### Prognostic risk model validation

**Fig. 4:**
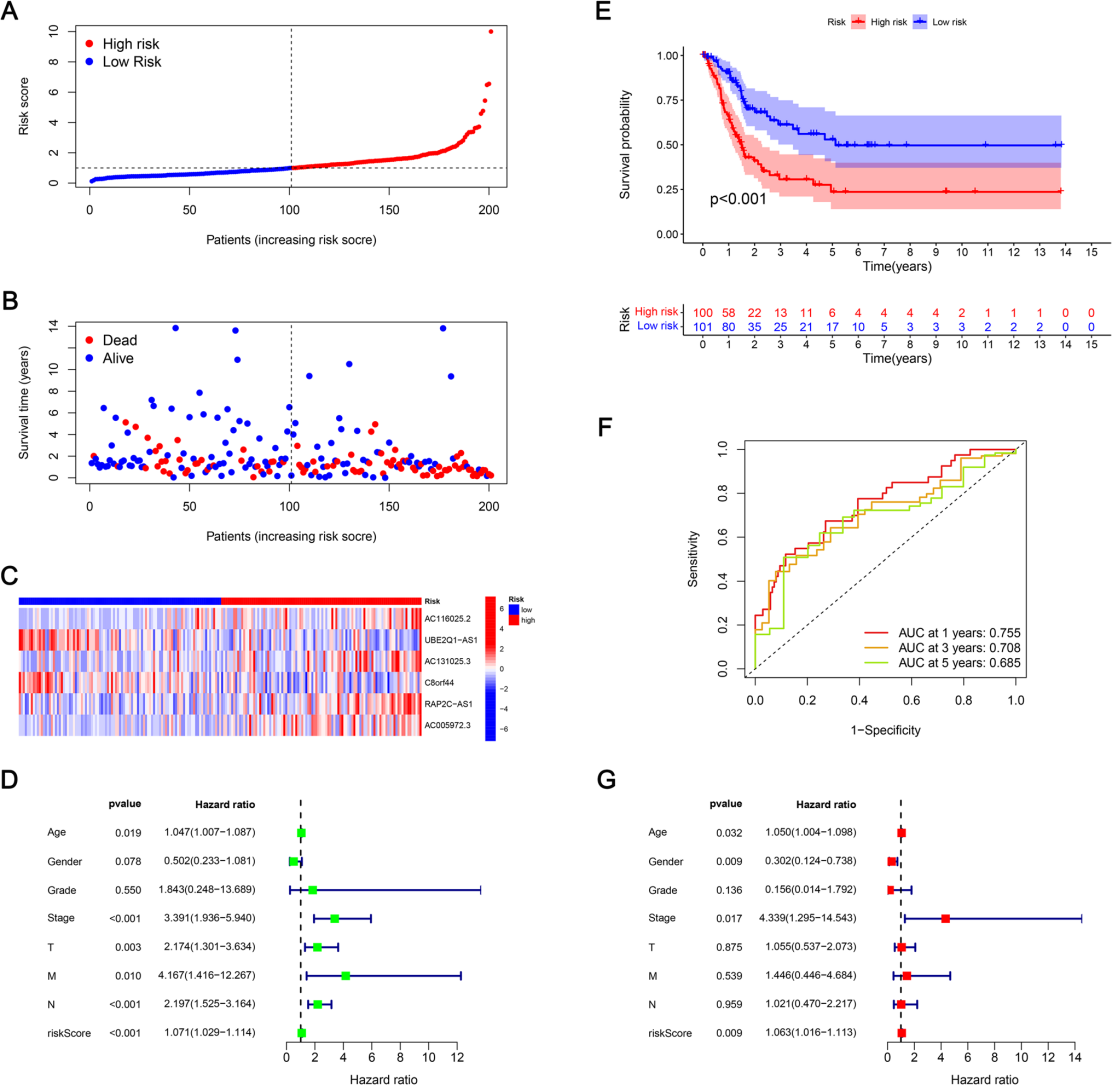
Prognostic value of mitochondrial-related lncRNA prognostic model in the training set. **A** RSs for mitochondrial-related lncRNA models in the training set. **B** Scatter plot for high- as well as low-risk survival status in the training set. **C** Heat map of 6 mitochondrial-related lncRNA in the training set. **D** The RS as well as clinicopathological characteristics as evaluated utilizing univariate regression analysis. **E** KM survival curves of OS between both risk group patients for the training set. **F** ROC curves for 1-, 3-, and 5-year periods were obtained by utilizing the model in the training set. **G** The RS as well as clinicopathological characteristics as evaluated utilizing multivariate regression analysis

**Fig. 5:**
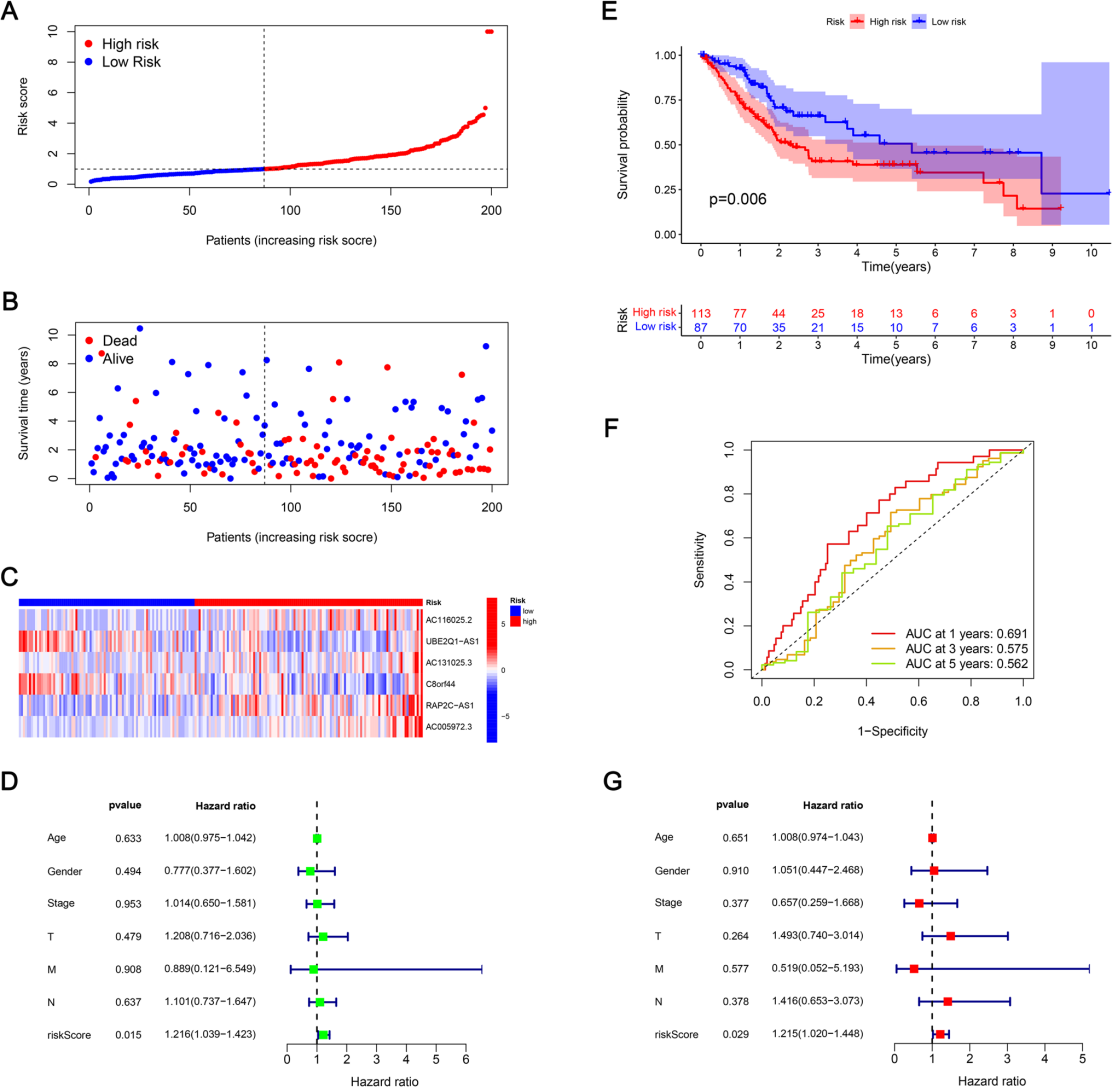
Prognostic value of mitochondrial-related lncRNA prognostic model in the testing set. **A** RSs for mitochondrial-related lncRNA models in the testing set. **B** Scatter plot of survival statuses in both risk groups in the testing set. **C** Heat map of—mitochondrial-related lncRNAs in the testing set. **D** Uni-Cox regression analysis of RS and clinicopathological characteristics. **E** KM survival curves of OS among both the risk groups for the testing set. **F** ROC curves for 1-, 3-, and 5-year periods were obtained utilizing the model in the testing set. **G** Multi-Cox regression analysis of RS and clinicopathological features

**Fig. 6:**
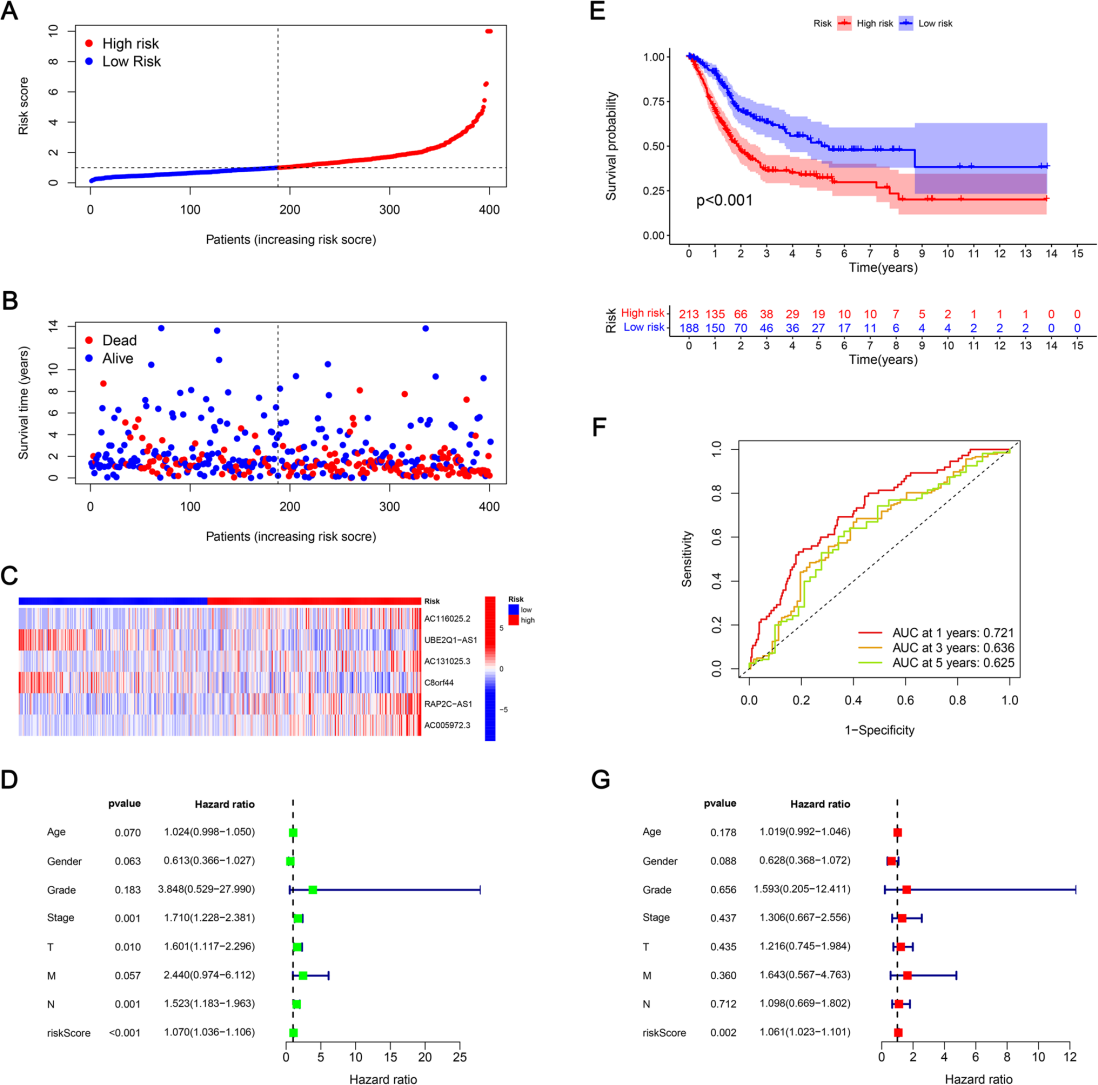
Prognostic value of mitochondrial-related lncRNA prognostic model in the entire set. **A** RSs for mitochondrial-related lncRNA models in the entire set. **B** Scatter plot of high- as well as low-risk survival status in the entire set. **C** Heat map of 6 mitochondrial-related lncRNAs in the entire set. **D** The RS as well as clinicopathological features as evaluated utilizing uni-Cox regression analysis. **E** KM survival curves of OS among patients of both risk groups for the entire set. **F** ROC curves for 1-, 3-, and 5-year periods were obtained by utilizing the model in the entire set. **G** Multi-Cox regression analysis of RS and clinicopathological features

For assessing the prognosis capacity of the developed model, it was divided into 3 sets: training, testing, and entire sets, and we then calculated the RS of each patient according to the RS formula and classified patients into two risk groups (low and high) as per the median risk value Group. The R software's "survival" package has been used to compare the RS distribution, survival status, survival time, and expression profiles of the lncRNAs related to low- as well as high-risk groups across the three concentrations. In the training set, the RS curve (Fig. 4A), survival status map (Fig. 4B), and heat map of 6 mitochondrial-related lncRNAs (Fig. 4C), OS of patients between both risk groups were plotted. The KM survival curve (Fig. 4E) constructed from these results indicated the mortality rate of patients in HRG was considerably higher than those in LRG. It should be noted that as the RS increased, the mortality rate of patients also increased, and the overall survival time of patients in HRG tended to decrease. The areas under the ROC curve (AUC) at 1, 3, and 5 years were 0.755, 0.708, and 0.685, respectively (Fig. 4F). Furthermore, the BLCA prognosis predictors were screened via univariate Cox regression analysis where age, stages (T, M, N), as well as RS, were all found to be related to BLCA prognosis (Fig. 4D). However, multivariate Cox regression analysis outcomes implied that age, sex, stage, and RS are independent predictors of BLCA prognosis (Fig. 4G). The RS curve (Fig. 5A), survival state map (Fig. 5B), and heat map of 6 mitochondrial-related lncRNA (Fig. 5C) in the testing set, the OS of patients in LRG, KM survival curve for the HRG (Fig. 5E) were constructed. The mortality rate of the patients in HRG has been found considerably higher than that of LRG. It is to be noted that with high RS, the mortality rate is higher, and the overall survival time of the patients in the HRG is shorter. The values for the areas under the ROC curve (AUC) were 0.691, 0.575, and 0.562 for 1-, 3-, and 5-year periods, respectively (Fig. 5F). Moreover, the predictors of BLCA prognosis were identified utilizing univariate Cox regression analysis. The results revealed an association of the RS with BLCA prognosis (Fig. 5D). Also, as per the findings of univariate Cox regression analysis, it was concluded that the RS served as an independent predictor for BLCA prognosis (Fig. 5G). For the entire set, the RS curve (Fig. 6A), survival status map (Fig. 6B), a heat map of six mitochondrial-related lncRNA genes (Fig. 6C), and OS of patients between both risk groups were plotted. The KM survival curve (Fig. 6E) constructed from these results indicated the mortality rate of patients in HRG is considerably higher than in HRG. It is worth noting that patients with higher RSs exhibited higher mortality rates and shorter overall survival times, particularly those in the HRG. The AUC values were found as 0.721, 0.636, and 0.625 for 1-, 3-, and 5-year periods, respectively (Fig. 6F). Also, the BLCA prognosis predictors were screened via univariate Cox regression analysis, and stage, T stage, N stage, and RS were all found to be related to BLCA prognosis (Fig. 6D). However, the results of multivariate Cox regression analysis implied that RS were independent predictors of BLCA prognosis (Fig. 6G).The prognostic risk model effectively stratifies BLCA patients based on survival outcomes. High-risk patients may exhibit more aggressive tumor behavior and poorer responses to conventional treatments, underscoring the need for targeted therapeutic strategies.

### Clinical evaluation of mitochondrial-related lncRNA prognostic signature

**Fig. 7:**
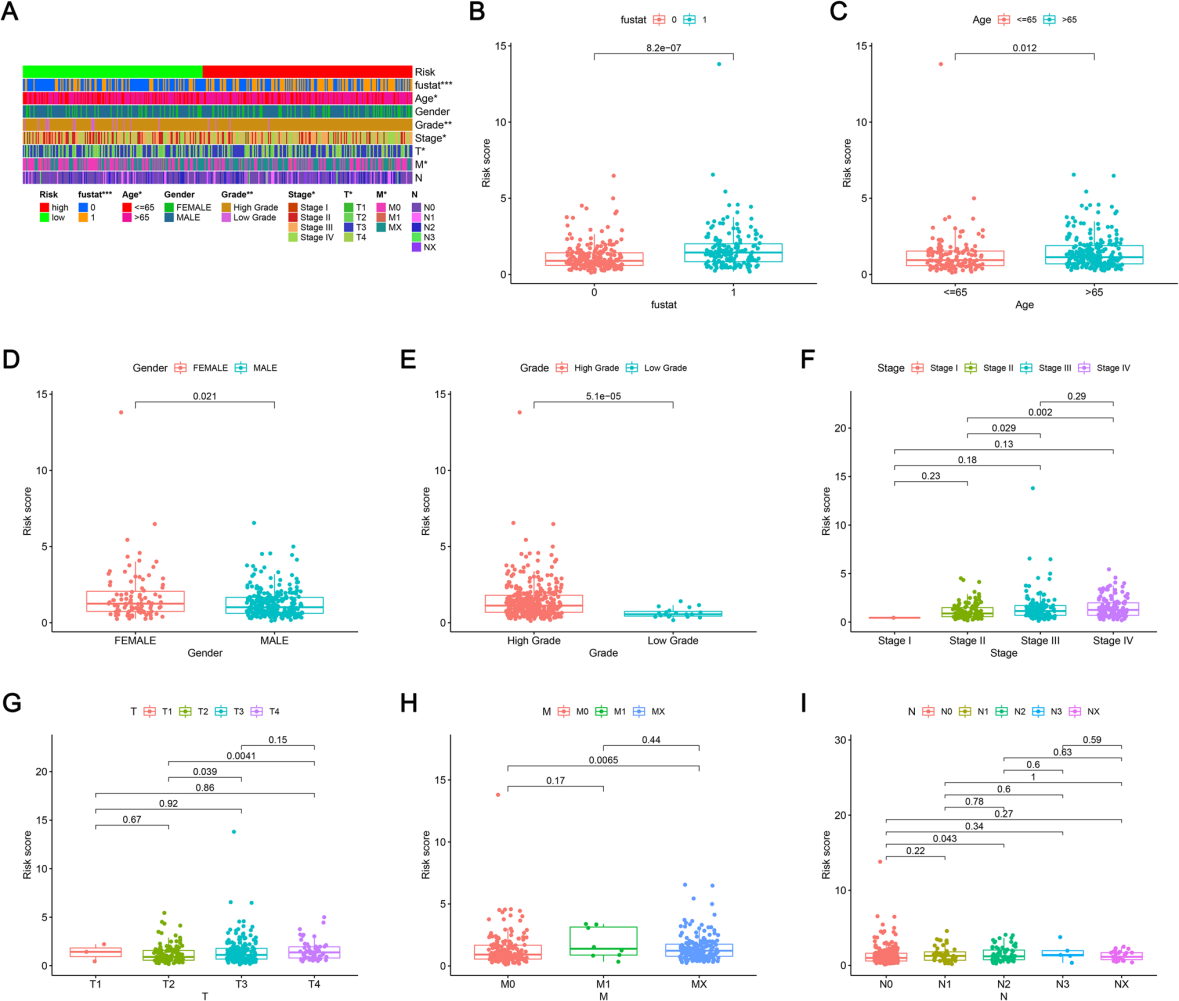
Clinical correlation of RSs utilizing clinicopathological features of BLCA patients. **A** Heatmap outlining common clinical characteristics, **B** Survival status (P < 0.001), **C** Age, **D** Gender, **E** Tumor grade, **F** Clinical stage, **G** T stage, **H** M phase, **I** N stage

A heat map (Fig. 7A) illustrating the correlation between RSs and clinical indicators was generated utilizing the Wilcoxon signed-rank test. From the map, it is evident that the survival status (Fig. 7B), age (Fig. 7C), tumor grade (Fig. 7E), clinical stage (Fig. 7F), T stage (Fig. 7G), and M stage (Fig. 7H) was significantly linked to the RS (P < 0.001). Conversely, gender (Fig. 7D) and N stage (Fig. 7I) did not exhibit any notable correlation with the RS (P > 0.05). The correlation of RS with adverse clinical features reinforces the model's relevance in predicting BLCA prognosis. High-risk patients may benefit from more intensive monitoring and personalized treatment approaches to improve outcomes.

3.5 PCA verifying the capability of the mitochondrial-related lncRNA signature to establish groups effectively

**Fig. 8:**
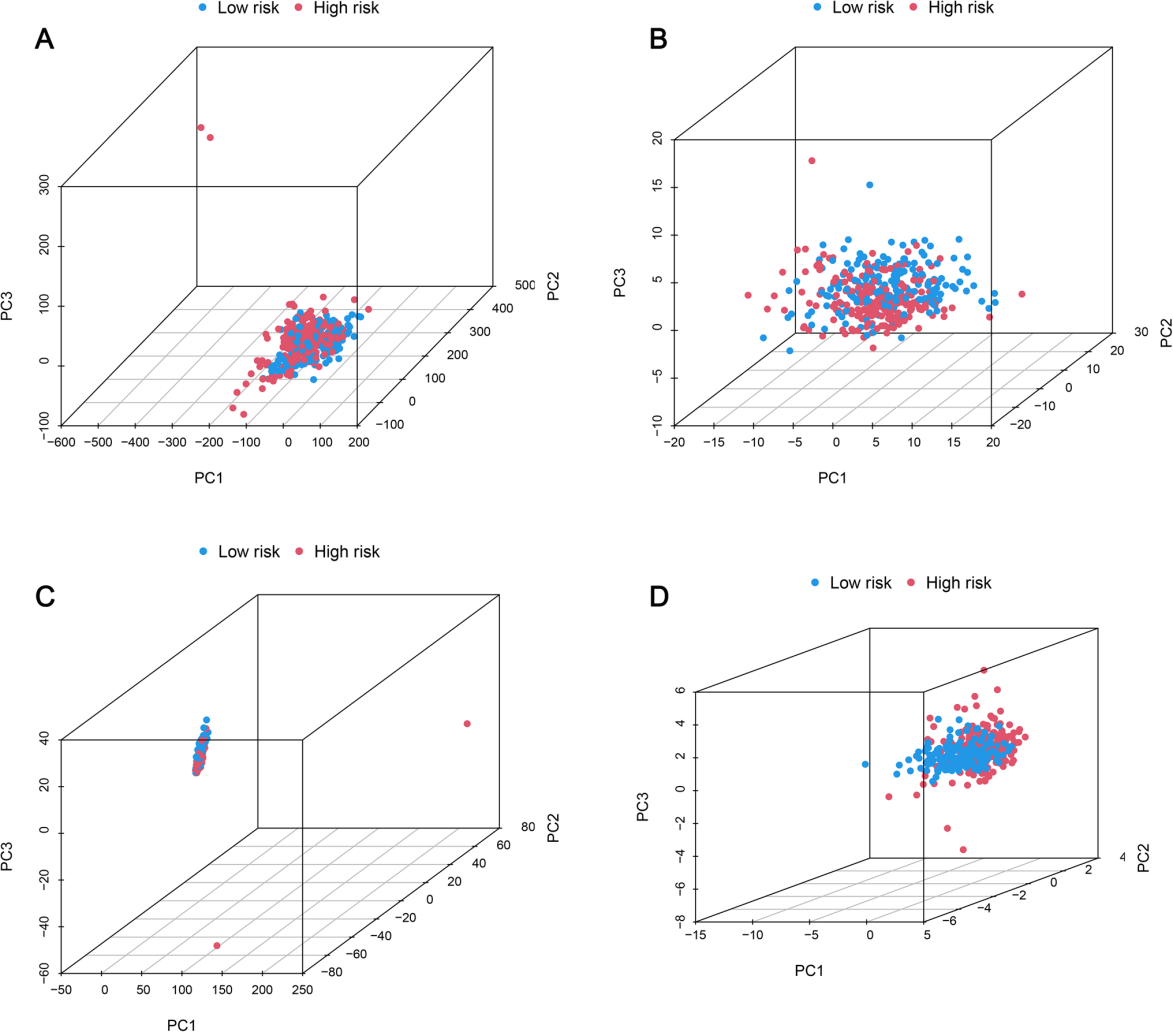
Following profiles were constructed based on the PCA of HRG and LRG. **A** Whole gene expression profile. **B** Mitochondrial-related gene expression profile. **C** Mitochondrial-related lncRNA expression profile. **D** Mitochondrial-related lncRNA prognostic signature expression profile for both risk groups

PCA revealed expression patterns for the whole genome (Fig. 8A), mitochondrial-related genes (Fig. 8B), mitochondrial-related lncRNA (Fig. 8C), and mitochondrial-related lncRNA prognostic signature for both risk groups (Fig. 8D). Figures 8A–C illustrate that both low-risk groups are widely dispersed in their distribution. Nonetheless, this model's findings reveal that the prognostic signature of mitochondrial-related lncRNAs exhibits distinct expression profiles for both risk groups (Fig. 8D). Therefore, these results demonstrate the capability of prognostic signature to successfully differentiate between LRG and HRG.

### GSEA and correlation analysis of prognostic risk model and tumor-infiltrating immune cells

**Fig. 9:**
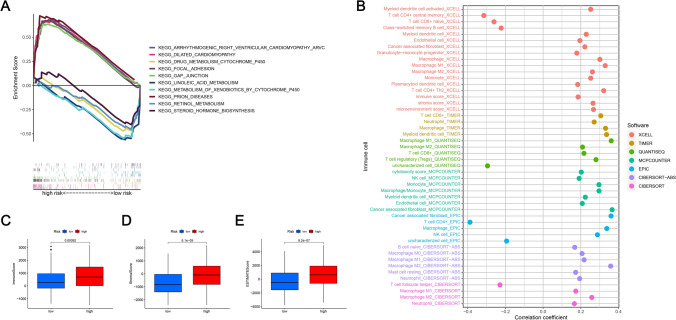
Prediction of tumor immunotherapy. **A** GSEA identified the top 5 pathways that exhibited considerable enrichment in both risk groups. **B** Association of tumor-infiltrating immune cells with prognostic signatures based on seven known algorithms. **C** Differences in immune scores between HRG and LRG. **D** Variations of stromal score between HRG and LRG. **E** ESTIMATE score variations between HRG and LRG

GSEA revealed a variety of mitochondrial and BLCA-related pathways, most of which are the prognostic marker regulators of mitochondrial-related lncRNAs, including drug metabolism cytochrome P450, linoleic acid metabolism, metabolism of xenobiotics by 450, retinol metabolism, and steroid hormone biosynthesis were considerably enriched in the LRG. The 5 pathways of arrhythmogenic right ventricular cardiomyopathy arvc, dilated cardiomyopathy, focal adhesion, gap junction, and prion diseases were found considerably enriched in the HRG (Fig. 9A). The relationship between prognostic features and tumor immune-infiltrating cells was evaluated utilizing PCA using seven algorithms and the results are illustrated via lollipops (Fig. 9B). The immune, stromal as well as estimate scores for each BLCA sample were computed via the ESTIMATE algorithm. Compared with the LRG, higher immune, stromal as well as estimate scores were observed in the HRG (P < 0.001), indicating different degrees of immune cell infiltration occurring in both risk groups. The boxplots showing variations in the number of tumor-infiltrating immune cells between both risk groups were constructed (Fig. 9C–E). The enriched pathways provide insights into the metabolic and signaling alterations in BLCA, highlighting potential therapeutic targets. The differences in immune cell infiltration between risk groups suggest that immune-related lncRNAs play a critical role in tumor microenvironment modulation and patient prognosis.

### Correlation between the prognostic risk model and ICIs

**Fig. 10:**
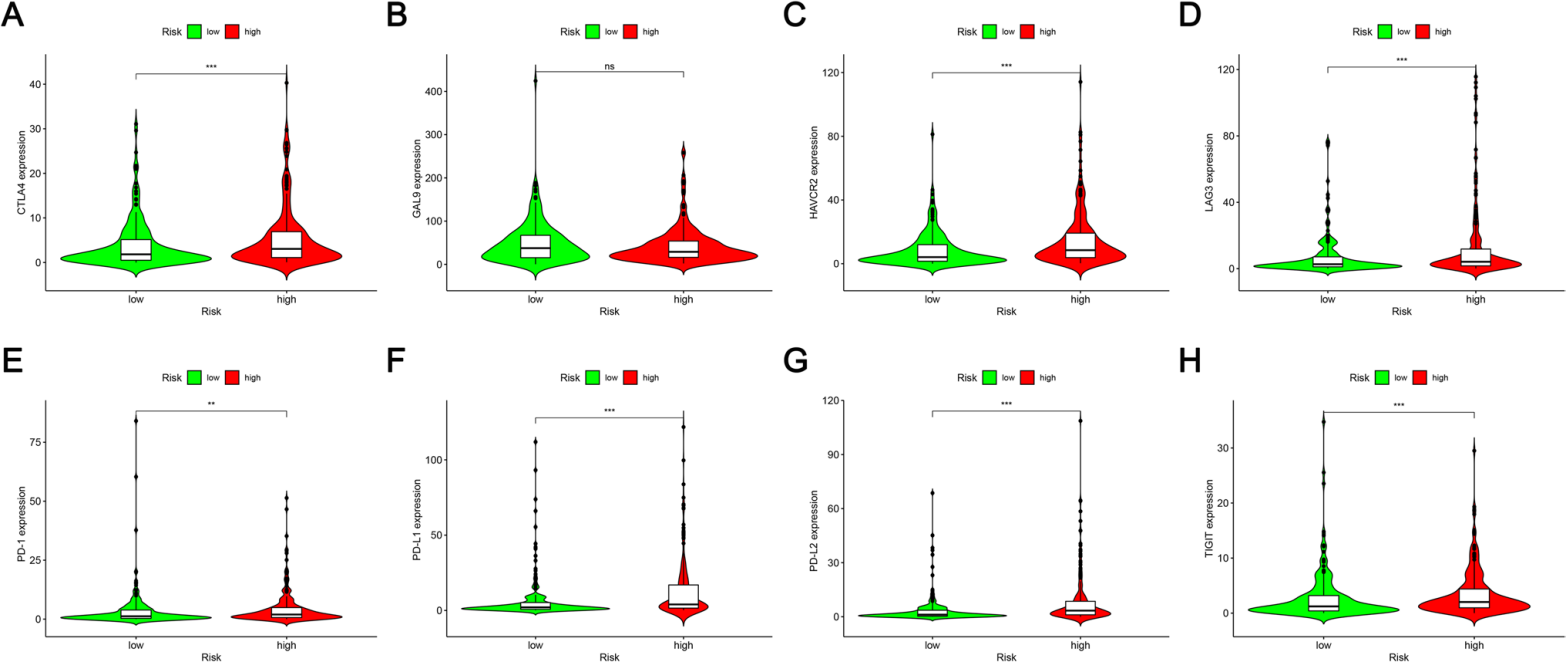
Relationship between prognostic features and the expression criteria of ICIs. **A** CTLA4 (P < 0.001), **B** GAL9 (P > 0.05), **C** HAVCR2 (P < 0.001), **D** LAG3 (P < 0.001), **E** PD-L1 (P < 0.01), **F** PD-L1 (P < 0.001), **G** PD-L2 (P < 0.001), and **H** TIGIT (P < 0.001)

The application of ICIs is one of the important potential treatments for BLCA. Hence, the correlation between the prognostic risk model and ICIs has been studied. High-RS and CTLA4 (P < 0.001; Fig. 10A), HAVCR2 (P < 0.001; Fig. 10C), LAG3 (P < 0.001; Fig. 10D), High expression of PD-L1 (P < 0.01; Fig. 10E), PD-L1 (P < 0.001; Fig. 10F), PD-L2 (P < 0.001; Fig. 10G), TIGIT (P < 0.001; Fig. 10H) were significantly correlated positively, but GAL9 was not varied considerably by risk group (P > 0.05; Fig. 10B). The association with immune checkpoints suggests that high-risk BLCA patients may benefit from immunotherapy, providing a rationale for incorporating immune checkpoint blockade in treatment plans for these patients.

### Analyzing the association between the prognostic risk model and targeted drug sensitivity

**Fig. 11:**
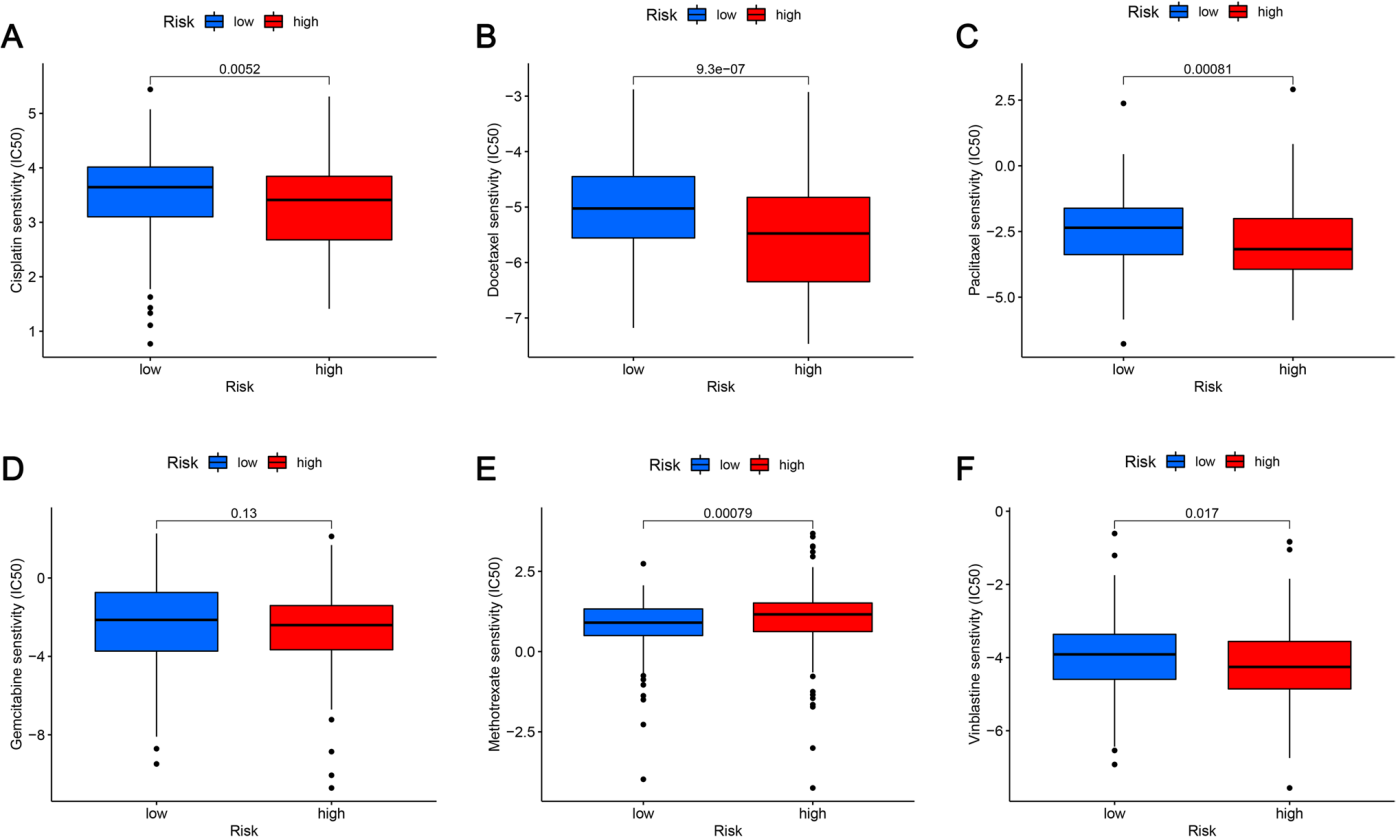
Relationship between chemotherapy drugs and risk model in patients with BLCA. In comparison to the LRG, individuals in the HRG exhibited a greater level of sensitivity to **A** cisplatin, **B** docetaxel, **C** paclitaxel, **D** gemcitabine, **E** methotrexate, and **F** vinblastine. **G** No significant differences in sensitivity were observed between the two groups regarding the drug gemcitabine

Targeted drugs are the most important primary treatment for advanced BLCA. The group at high risk was discovered to have an association with low IC50s. for cisplatin (Fig. 11A), docetaxel (Fig. 11B), paclitaxel (Fig. 11C), methotrexate (Fig. 11E), and vinblastine (Fig. 11F), suggesting that the prognostic risk model developed in this study can predict these drug sensitivities. Contrarily, the IC50 value of gemcitabine (Fig. 11D) didn’t display any remarkable variations between both risk groups. The differential drug sensitivity between risk groups underscores the importance of personalized chemotherapy regimens based on the prognostic risk model, potentially improving therapeutic efficacy and minimizing adverse effects.

## Discussion

Although BLCA treatment strategies such as tumor resection, chemotherapy, and radiotherapy have made great progress, the etiology and clinicopathological manifestations of a highly heterogeneous malignant tumor like BLCA vary among patients. Therefore, the effect of these BLCA treatments is not always satisfactory in all patients. A reliable BLCA prognostic model urgently needs to be screened out for risk stratification and prognostic analysis of BLCA. Accumulating evidence shows that lncRNAs play a crucial role in the occurrence, development, and metastasis of BLCA, and demonstrate their potential as a novel biomarker. Studies have revealed that lncRNA-RMRP may enhance the proliferation, migration as well as invasion of BLCA cells [6]. By modulating E2F1, lncRNA-SLC16A1-AS1 can enhance metabolic reprogramming in BLCA [19]. Additionally, lncRNA TUC338 is identified as a potential diagnostic biomarker for BLCA [20]. Furthermore, prognostic models based on lncRNA expression have been validated for predicting the survival or recurrence of BLCA [21]. However, it is still unclear whether the mitochondrial-associated lncRNA model can successfully predict BLCA prognosis.

In the current study, we identified six mitochondrial-associated lncRNAs (AC116025.2, UBE2Q1-AS1, AC131025.3, C8orf44, RAP2C-AS1, AC005972.3) that were used to form an independent prognostic risk model for BLCA. Each of these lncRNAs has potential biological roles that could influence BLCA pathology. Previous studies have shown that the upregulation of AC116025.2 affects immune cell infiltration and is associated with hepatocellular carcinoma prognosis [22]. Its role in modulating the immune microenvironment may also be critical in BLCA, suggesting that AC116025.2 could influence tumor-immune interactions and contribute to tumor progression and patient outcomes. A copper apoptosis-related lncRNA prognostic model including UBE2Q1-AS1 has been associated with BLCA prognosis [23]. UBE2Q1-AS1 may play a role in regulating apoptotic pathways, which are crucial for tumor cell survival and resistance to therapies. Although specific studies on AC131025.3 are limited [7], it is possible that this lncRNA could be involved in mitochondrial function and metabolic regulation, given its association with mitochondrial genes. Dysregulation of mitochondrial function is a known factor in cancer development and progression. Like AC131025.3, C8orf44 may also be involved in mitochondrial regulation. Mitochondria are essential for energy production and metabolic homeostasis, and their dysfunction can lead to cancer progression. RAP2C-AS1 has been reported to be substantially related to overall survival in esophageal cancer patients [24]. Its involvement in BLCA could similarly influence survival outcomes by affecting cellular processes such as proliferation and apoptosis. While specific roles of AC005972.3 in cancer are not well-documented, its inclusion in the prognostic model suggests it may have significant biological functions in BLCA, potentially related to mitochondrial regulation and cellular metabolism [8]. By exploring these biological mechanisms, we provide a more comprehensive understanding of how these lncRNAs contribute to BLCA pathology. These insights enhance the study's impact by linking the prognostic model to underlying molecular processes, which can inform future research and therapeutic strategies.

Moreover, the clinicopathological characteristics were combined with multi-Cox regression analysis. The prognostic risk model showed a significant correlation with BLCA patients' survival status, age, stage, and TNM stage. The RS was also identified as an independent predictor of BLCA, accurately predicting survival rates for 1-, 3-, and 5-year periods. The study found that the developed model had good specificity and sensitivity for overall survival, as shown by the AUC values. Specifically, the 1-, 3-, and 5-year AUC values were 0.755, 0.708, and 0.685 for the total set; 0.691, 0.575, and 0.562 for the training set; 0.721, 0.636, and 0.625 for the test set. Through the prognostic risk model, patients were classified into LRG and HRG as per computed median RS. Moreover, KM analysis, GSEA, and IC50 prediction were performed, and the OS of patients in the LRG was found to be considerably higher than patients in the HRG. GSEA was carried out to explore the biological functions of the prognostic model features, revealing multiple mitochondrial and tumor-related pathways, most of which were the prognostic marker regulators of mitochondrial lncRNAs. The research findings suggest that there are distinct immune microenvironments present in both risk groups. Compared with the LRG, higher immune stromal as well as estimate scores were observed for the HRG (P < 0.001). This indicates that different risk groups experience different degrees of immune cell infiltration, which leads to different prognoses and responses to immunotherapy.

Fifty percent of BLCA patients relapse post-radical surgery and often exhibit distant metastases. Although immunotherapy provides a promising new treatment scope for metastatic BLCA [25], only 20–30% of advanced BLCA patients respond to immunotherapy. Therefore, the exploration of potentially predictive biomarkers for immunotherapy requires urgent attention. The study revealed a considerable positive correlation between the HRG and increased expression levels of CTLA4, LAG3, PD-1, TIGIT, PD-L1, PD-L2, and TIM-3. The findings suggest that the prognostic risk model involving mitochondrial-associated lncRNA could potentially serve as a predictive tool for immunotherapy response in BLCA. Chemotherapy drugs are the most important primary treatment for advanced BLCA to date. Even though neoadjuvant chemotherapy has been reported as a treatment option for BLCA patients before undergoing radical cystectomy, it is crucial to note that not all BLCA patients may respond to chemotherapy [26]. Early prediction of the chemotherapy response can significantly reduce side effects. The data reveal that the HRG demonstrated higher sensitivity to cisplatin, docetaxel, paclitaxel, methotrexate, and vinblastine compared to the LRG. This data conveys that the risk prognostic model developed in this study may have the potential to predict the susceptibility of BLCA to these chemotherapeutic drugs.

In summary, the current study established that a prognostic risk model constructed based on mitochondrial lncRNA can accurately predict the survival prognosis of BLCA patients and is an independent prognostic factor for BLCA. Based on the prognostic risk model, patients with bladder cancer can be categorized into high- or low-risk groups, which is beneficial to guide individualized BLCA treatment. This study also demonstrated that the selected six mitochondrial-related lncRNAs are potential prognostic and diagnostic biomarkers of BLCA, as well as potential therapeutic targets of the disease. Thus, the findings of this study suggest the application potential of the six aforementioned lncRNAs in predicting immune status and chemotherapy response in BLCA. Despite certain valuable findings, it is important to note that this study has certain limitations. First, the study is retrospective in nature, using data from the TCGA database, which lacks detailed clinical history and treatment information. Second, the study was based on 401 patients listed in the publicly available TCGA database. The limited sample size may hamper the reliability of the predictive performance of the model. Therefore, multi-centered and prospective studies on a larger scale are required to validate these results before the mitochondrial lncRNA prognostic models are applied in the clinic.

Implementing this prognostic model in a clinical setting involves several practical steps and challenges. First, RNA sequencing or similar high-throughput transcriptomic technologies must be incorporated into routine diagnostic workflows to measure the expression levels of the six identified lncRNAs. This requires investment in equipment, training for laboratory personnel, and establishing standardized protocols. Development and validation of clinical-grade assays specific to the 6 lncRNAs are crucial, and collaboration with diagnostic companies and regulatory bodies will be necessary. Integration of the prognostic model into electronic health record systems will facilitate seamless use of risk scores in clinical decision-making. Challenges include ensuring data privacy and security, the cost of implementing advanced diagnostic tools, and resistance to adopting new technologies and workflows among healthcare professionals. Continuous education and demonstration of the model's clinical utility through pilot studies and clinical trials will be vital in overcoming resistance.

In conclusion, while there are challenges in implementing the mitochondrial-associated lncRNA prognostic model in clinical settings, the potential benefits in terms of personalized treatment and improved patient outcomes make it a worthwhile endeavor. Ongoing research, technological advancements, and collaborative efforts will be key to successfully integrating this model into routine clinical practice.

## Data Availability

The study's datasets can be found at [https://portal.gdc.cancer.gov/].
